# The Metabolic-Immune Axis: Amino Acids and Immune Cell Dynamics in Mammary Gland Development and Remodeling

**DOI:** 10.7150/ijbs.138760

**Published:** 2026-07-20

**Authors:** Xiangyang Ye, Ziwei Xu, Yating Chen, Yusheng Lu, Li Wang, Hao Xiao

**Affiliations:** 1 State Key Laboratory of Swine and Poultry Breeding; Key Laboratory of Animal Nutrition and Feed Science in South China, Ministry of Agriculture and Rural Affairs; Guangdong Provincial Key Laboratory of Animal Breeding and Nutrition; Institute of Animal Science, Guangdong Academy of Agricultural Sciences, Guangzhou 510640, China.; 2 Institute of Agricultural Resources and Environment, Guangdong Academy of Agricultural Sciences, Guangzhou 510640, China.

**Keywords:** mammary gland remodeling, immune cells, amino acid metabolism

## Abstract

The mammary gland undergoes tightly coordinated cycles of growth, differentiation and regression, which require precise communication between epithelial and stromal cells. Immune cells, whether tissue-resident or recruited, are well-established regulators of mammary development, which is orchestrated by hormones, cytokines, growth factors, microorganisms and nutritional signals. As essential building blocks for protein synthesis, amino acids modulate systemic metabolic homeostasis and influence the development, differentiation, and functional capacity of immune cells. However, the relationship between amino acid metabolism and immune regulation during mammary gland remodeling is not well understood. Here, we review the current understanding of the multiple biological factors underlying mammary gland development and remodeling, the composition and function of mammary immune cells across developmental stages, dynamic changes in mammary gland amino acids and transporters, and amino acid metabolism in the mammary gland and its immunomodulatory mechanisms. This improves our understanding of normal mammary biology and its implications for pathological conditions, such as lactation insufficiency and breast cancer.

## 1. Introduction

Mammary gland development is a dynamic process spanning the embryonic stage, puberty, pregnancy, lactation, and involution^1^. Initial formation occurs during embryogenesis. Puberty drives rapid ductal expansion and branching. In murine models, terminal end buds (TEBs) emerge at the distal ends of the ducts and invade the surrounding adipose stroma, thereby facilitating further development. Under hormonal regulation, pregnancy induces lobuloalveolar proliferation and differentiation to form a functional, milk-secreting gland by term^2^, which is activated during lactation^3^. After weaning, widespread apoptosis of the secretory epithelium is triggered by involution, followed by stromal repopulation by fibrous tissue and adipocytes^4^. This process is orchestrated by a complex interplay of systemic hormones, local paracrine signals, the extracellular matrix (ECM), immune cells, stem cells and their lineage commitment, transcriptional networks, and nutritional cues. Estrogen and progesterone, along with prolactin and growth hormone, drive ductal elongation and alveolar differentiation^5^; locally synthesized growth factors such as Insulin-like growth factor 1 (IGF-1), Epidermal growth factor (EGF), and Transforming growth factor beta (TGF-β) mediate reciprocal epithelial-stromal signaling^6^; and immune cells, including macrophages, eosinophils, and T cells, actively participate in tissue remodeling and stem cell regulation throughout developmental stages^7^. The recruitment and function of these immune cells are in turn influenced by hormonal fluctuations, microbial signals, and local metabolic cues^8^.

Amino acids (AAs) are fundamental to protein synthesis and regulate metabolic pathways that maintain systemic homeostasis^9^. Beyond this classic role, they also function as critical signaling molecules that govern immune cell development, differentiation, and effector activity^10^. Notably, amino acid transporters initiate nutrient-sensing that leads to mechanistic target of rapamycin (mTOR) activation, which coordinates cellular energy metabolism and promotes cell proliferation^11^. Recent advances in immunometabolism have emphasized the complex relationship between amino acid availability and immune regulation, providing a compelling model for understanding tissue adaptation^12^, ^13^.

Immune cells play an essential role in remodeling mammary tissue at different stages of development. Amino acids act as vital signaling molecules that govern immune cell metabolism and function. However, the specific ways in which they regulate mammary immune cells within the tissue microenvironment remain largely unknown. Here, we summarize the current understanding of the multiple biological processes underlying mammary gland development and remodeling, outlining major immune cell functions in mammary morphogenesis and homeostasis, immune dynamics and stage/specific amino acid changes in blood and milk, and how amino acids regulate mammary immune cell metabolism, polarization, and function. Together, these findings reveal a metabolic-immune framework where amino acids act as key regulators, coordinating mammary tissue remodeling and functional adaptation across reproductive cycles.

## 2. Main biological factors involved in mammary gland development and remodeling

**Figure 1:**
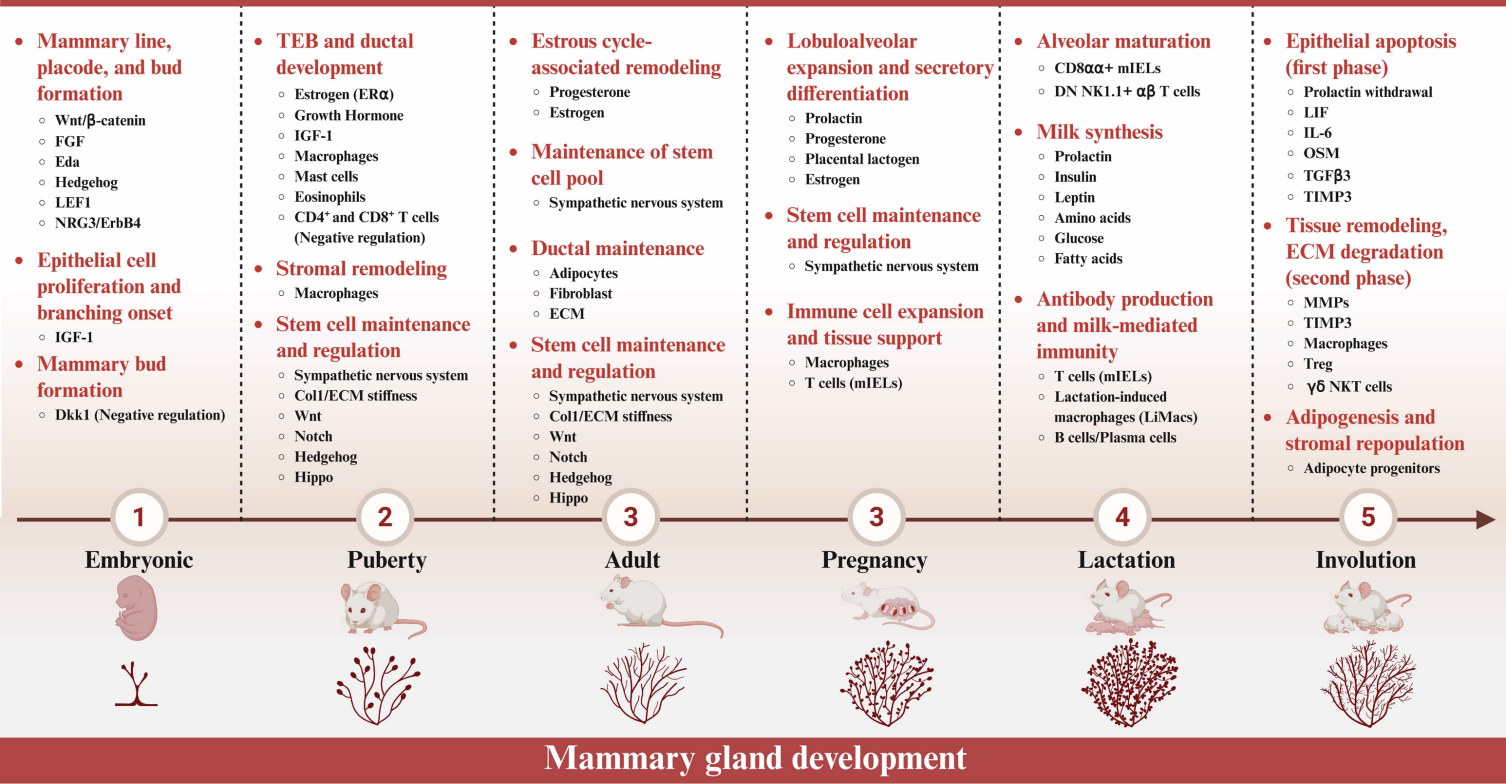
** Stage-specific regulatory mechanisms of mouse mammary gland development.** This schematic summarizes the key regulatory factors and signaling pathways that control mouse mammary gland development across six stages: embryonic, puberty, adult, pregnancy, lactation, and involution. (Created in BioRender, https:/BioRender.com).

Mammary gland development proceeds through a series of precisely orchestrated stages, each of which is controlled by a complex network of systemic hormones, local paracrine signals, extracellular matrix remodeling and mammary gland cells and so on. Among experimental models, the mouse provides the most comprehensive mechanistic data across all developmental stages, making it the reference system for our schematic overview (Figure 1). In this chapter, we briefly describe the current understanding of the multiple biological factors underlying mammary gland development and remodeling, emphasizing how they coordinate tissue remodeling across different reproductive stages.

### 2.1 Systemic hormones

Systemic hormones orchestrate the structural remodeling of the mammary gland throughout the reproductive cycle. Given that the hormonal regulation of mammary gland development has been extensively reviewed elsewhere, we provide only a brief overview here, focusing on the key hormones that orchestrate structural remodeling across reproductive stages^14^. During puberty, estrogen acts through estrogen receptor alpha (ERα)-positive luminal epithelial cells to drive ductal elongation via TEB formation and invasion, with growth hormone (GH) and insulin-like growth factor 1 (IGF-1) providing synergistic proliferative signals^15^. During pregnancy, progesterone promotes side branching and alveolar budding, and its effects on mammary epithelial subset expansion are mediated through receptor activator of nuclear factor kappa-B (RANK) signaling^16^. Concurrently, progesterone suppresses premature lactogenic differentiation, and its withdrawal at parturition relieves this inhibition, unleashing prolactin signaling to initiate lactogenesis II and copious milk secretion^15^, ^17^, ^18^. Prolactin, upon binding to its receptor PRLR, activates Janus kinase 2 (JAK2), which phosphorylates signal transducer and activator of transcription 5 (STAT5) and promotes its nuclear translocation to initiate milk protein gene expression, including β-casein^19^. Prolactin also modulates luminal hormone-responsive (LumHR) cell behavior through dynamic changes in its receptor signaling, positioning prolactin as a critical switch in regenerative mammary remodeling^20^.

### 2.2 Paracrine signaling and extracellular matrix remodeling

Local paracrine signals and ECM remodeling have been extensively characterized in mammary development^21^. Here we briefly summarize the key mediators and their stage-specific functions.

Paracrine signals are locally secreted factors that mediate communication between adjacent cells. Within the epithelium, members of the EGF family, such as amphiregulin (AREG) and heparin-binding EGF-like growth factor (HB-EGF), mediate estrogen action: ERα-positive cells secrete these ligands to activate epidermal growth factor receptor (EGFR) on adjacent ERα-negative epithelial progenitors, driving cap cell proliferation and branching morphogenesis^22^. Between epithelium and stroma, embryonic Hedgehog signaling induces mesenchymal fibroblast growth factor 10 (FGF10), which acts on epithelial fibroblast growth factor receptor 2b (FGFR2b) to promote mammary bud formation^23^, ^24^; during puberty, stromal hepatocyte growth factor (HGF)-c-Met signaling drives ductal branch extension^25^, ^26^.

The ECM is a dynamic network of proteins and polysaccharides that provide structural support and actively regulate cell behavior. Fibroblast-derived matrix metalloproteinases (MMPs) remodel the ECM to clear physical barriers and release ECM-bound growth factors, facilitating ductal invasion through the fat pad during pubertal morphogenesis^27^. The integrin/ECM mechanosensing axis, mediated by integrins such as α6β1 and α2β1, transduces biochemical and mechanical cues (e.g., ECM stiffness) via focal adhesion kinase (FAK)/Src and Yes-associated protein (YAP)/transcriptional coactivator with PDZ-binding motif (TAZ) to regulate cell proliferation, differentiation, and polarity. Notably, ECM stiffness modulates epithelial sensitivity to hormonal signals, establishing communication between endocrine cues and the physical microenvironment^28^.

Together, these paracrine and matrix-derived signals form an integrated local regulatory network that complements systemic hormonal control and coordinates mammary tissue remodeling across developmental stages.

### 2.3 Mammary gland cells

Multiple cell types, including stem cells, epithelial cells, fibroblasts, and immune cells, work together to drive the development and remodeling of mammary gland^29^. This section discusses the specific roles and mechanisms of these cell types in mammary development and tissue remodeling.

#### 2.3.1 MaSCs

Mammary stem cells (MaSCs) sustain mammary gland development, homeostasis, and regeneration across successive reproductive cycles. During puberty, MaSCs generate progenitors that drive ductal elongation; during pregnancy, they expand to support alveolar formation; after involution, a surviving subset ensures gland renewal for subsequent pregnancies^30^. Given that MaSCs have been thoroughly reviewed elsewhere^31^, we focus on their functional heterogeneity and the key signaling pathways that control their behavior during mammary gland remodeling.

MaSCs are functionally heterogeneous. Bipotent stem cells (e.g., CD34^-^CD200^+^; Bcl11b^+^) at the nipple region maintain long-term homeostasis and multi-cycle regeneration, whereas unipotent progenitors (e.g., CD34^+^CD200^-^; Sema3a^+)^ in terminal end buds respond rapidly to pregnancy for acute alveologenesis^32^. The behavior of these pools is governed by key signaling pathways. The Notch pathway directs basal-to-luminal commitment, while the Wnt/β-catenin pathway promotes self-renewal and progenitor expansion^33^. In addition, discoidin domain receptor 1 (DDR1)-runt-related transcription factor 1 (RUNX1) drives differentiation. However, its inhibition traps stem cells in a bipotent state, thereby blocking alveolar development^34^.

#### 2.3.2 Mammary epithelial cells

Mammary epithelial cells (MECs) are the primary structural and functional units of the mammary gland, directly executing ductal elongation, alveolar development, and milk secretion across reproductive stages^15^. As the ultimate effector layer of mammary tissue remodeling, MECs perceive hormonal and paracrine inputs and translate them into specific cellular responses. During pregnancy, MECs undergo alveolar differentiation and secretory maturation, a process that is fine-tuned by intrinsic regulators such as vang-like protein 2 (VANGL2), which provides negative feedback via nuclear translocation to prevent premature differentiation^35^. At the same time, MECs actively restrain fibroblast activation and excessive ECM remodeling through the receptor tyrosine kinase (RTK)/Sprouty axis, thereby preserving epithelial-stromal homeostasis^36^.

MECs also respond directly to the physical properties of their microenvironment. During puberty and early pregnancy, they promote ductal elongation in response to hyaluronic acid (HA)-enriched ECM; during mid- and late pregnancy, they adapt to increased collagen deposition and elevated matrix stiffness by committing to alveolar differentiation^37^. Thus, by integrating biochemical and physical cues, MECs directly facilitate mammary gland remodeling throughout all developmental stages.

#### 2.3.3 Fibroblasts

Fibroblast biology in the mammary gland has been extensively characterized; here we focus on their stage-specific functions in ECM remodeling and paracrine regulation during development and remodeling^38^. Fibroblasts actively regulate mammary epithelial morphogenesis by orchestrating ductal elongation, alveolar expansion, and post-lactational regression through dynamic ECM remodeling and paracrine signaling. During puberty, they construct a transient stromal sheath around TEBs, creating a permissive microenvironment by depositing collagen, fibronectin, and HA^37^. During pregnancy and lactation, they deposit extensive collagen, increasing matrix stiffness and activating integrin-FAK-YAP/TAZ mechanotransduction in epithelial cells, thereby driving proliferation and secretory differentiation^37^. Upon weaning, fibroblasts adopt a myofibroblast-like phenotype and secrete MMPs to degrade excess ECM, thereby promoting tissue regression. Throughout these stages, fibroblast function is tightly controlled by the RTK/Sprouty axis. Loss of Spry1/2/4 leads to RTK hyperactivation, excessive ECM remodeling, and disruption of epithelial architecture^36^. Thus, fibroblasts act as dynamic mediators that integrate physical and biochemical cues to coordinate epithelial behavior across the reproductive cycle, and their dysfunction contributes to the pathogenesis of breast disease.

#### 2.3.4 Immune cells

Immune cells play a crucial role in regulating the development of the mammary gland, extending their function beyond defending the host to actively coordinating tissue remodeling throughout postnatal stages. Macrophages, eosinophils, mast cells, T cells, and B cells infiltrate the gland in a stage-specific manner. They work alongside hormones, epithelial cells, and stromal cells to direct morphogenesis and homeostasis^29^. Rather than being a conventional immune response, this process is a form of 'sterile inflammation' driven by tissue expansion, metabolic stress, and cell death. These factors recruit and polarize immune cells to support stage-specific developmental outcomes^7^.

Despite their recognized roles at all major stages, our understanding of immune cell function in mammary development remains uneven. While their contributions to pubertal ductal elongation and post-lactational involution are relatively well understood, the mechanisms governing pregnancy and lactation are less well understood. These are stages of high replicative and metabolic demand. This is particularly true of the processes of macrophage polarization, T cell dynamics, and the signaling pathways, such as signal transducer and activator of transcription 3 (STAT3) and nuclear factor kappa-light-chain-enhancer of activated B cells (NF-κB), that mediate immune-epithelial crosstalk^7^. The following chapter provides a comprehensive, stage-by-stage account of their composition, localization, and functional contributions.

### 2.4 Nutritional regulation

Nutritional status profoundly influences mammary gland development and lactation capacity. The high metabolic demands of mammary epithelial proliferation, differentiation, and milk synthesis require an adequate supply of glucose, fatty acids, and amino acids. Glucose provides fundamental energy and carbon backbone. During lactation, glucose transporter type 1 (GLUT1) expression increases and is redistributed to the Golgi apparatus in response to low oxygen tension rather than classical hormones. This influences not only lactose synthesis, but also epithelial cell proliferation and metabolic adaptation 39. Fatty acids are equally indispensable, yet their effects vary markedly depending on their composition. n-3 polyunsaturated fatty acids (PUFAs) such as docosahexaenoic acid (DHA) activate G protein-coupled receptor 120 (GPR120)-cyclic adenosine monophosphate (cAMP)-exchange protein directly activated by cAMP (EPAC) signaling to promote epithelial morphogenesis^40^, whereas elevated palmitic acid suppresses mammary branching via cluster of differentiation 36 (CD36) palmitoylation and c-Jun N-terminal kinase (JNK)-extracellular signal-regulated kinase (ERK) activation^41^.

Unlike glucose and fatty acids, which primarily serve as energy sources and structural components, amino acids function as both building blocks and signaling molecules. Lysine activates the ERK1/2-cyclin-dependent kinase 1 (CDK1)-mTOR axis, promoting cell proliferation and β-casein synthesis^42^. Glutamine fuels O-GlcNAcylation (a post-translational modification involving the addition of O-linked β-N-acetylglucosamine to proteins) to modulate transcription factors essential for cell fate decisions^43^. BCAAs are catabolized to generate glutamate, linking nutrient sensing to cellular energetics^44^. Tryptophan metabolites activate hydrocarbon receptor (AhR), which interacts with the Wnt and Notch pathways in the mammary gland^45^, ^46^. Given that amino acids uniquely function as both precursors and signals, the following chapters will focus on their stage and species-specific changes in blood and milk, as well as their immunomodulatory roles within the mammary gland.

### 2.5 Microbial community

The impact of the microbial community on mammary gland development and remodeling is an emerging area of research in mammary biology. This is primarily mediated by the gut-mammary axis, a regulatory network integrating microbial signals with host hormonal, neural and immune pathways^47^. Current evidence suggests that microbial signals reach the mammary gland via several different routes. One such route is via the vagus nerve, which mediates communication along the gut-brain-mammary axis to regulate BDNF (brain-derived neurotrophic factor) secretion, which in turn influences mammary sensory nerve development and pubertal ductal branching^48^. Additionally, gut-resident bacteria and their extracellular vesicles can translocate to mammary tissue via dendritic cells or the lymphatic system. There, they activate NF-κB signaling through pattern recognition receptors such as Toll-like receptor 2 (TLR2) and Toll-like receptor 4 (TLR4), thereby participating in local immune surveillance^49^.

Although the mammary gland itself harbors a low biomass of microbes, this community is functionally active. Beneficial commensal bacteria, such as *Lactobacillus* and *Bifidobacterium*, contribute to the integrity of the epithelial barrier and local immune homeostasis by secreting immunomodulatory metabolites, including short-chain fatty acids^49^. During involution, systemic low-grade inflammation associated with dysbiosis can delay transition of macrophages from the M1 to M2 state and impair autophagy-related (ATG) protein-mediated efferocytosis, leading to aberrant ECM degradation and fibrosis 50, 51. However, as this field is still in its early stages, the underlying mechanisms, particularly the specific microbial signals and their receptors involved in mammary remodeling, remain to be fully elucidated.

## 3. Composition and function of mammary immune cells across developmental stages

**Figure 2:**
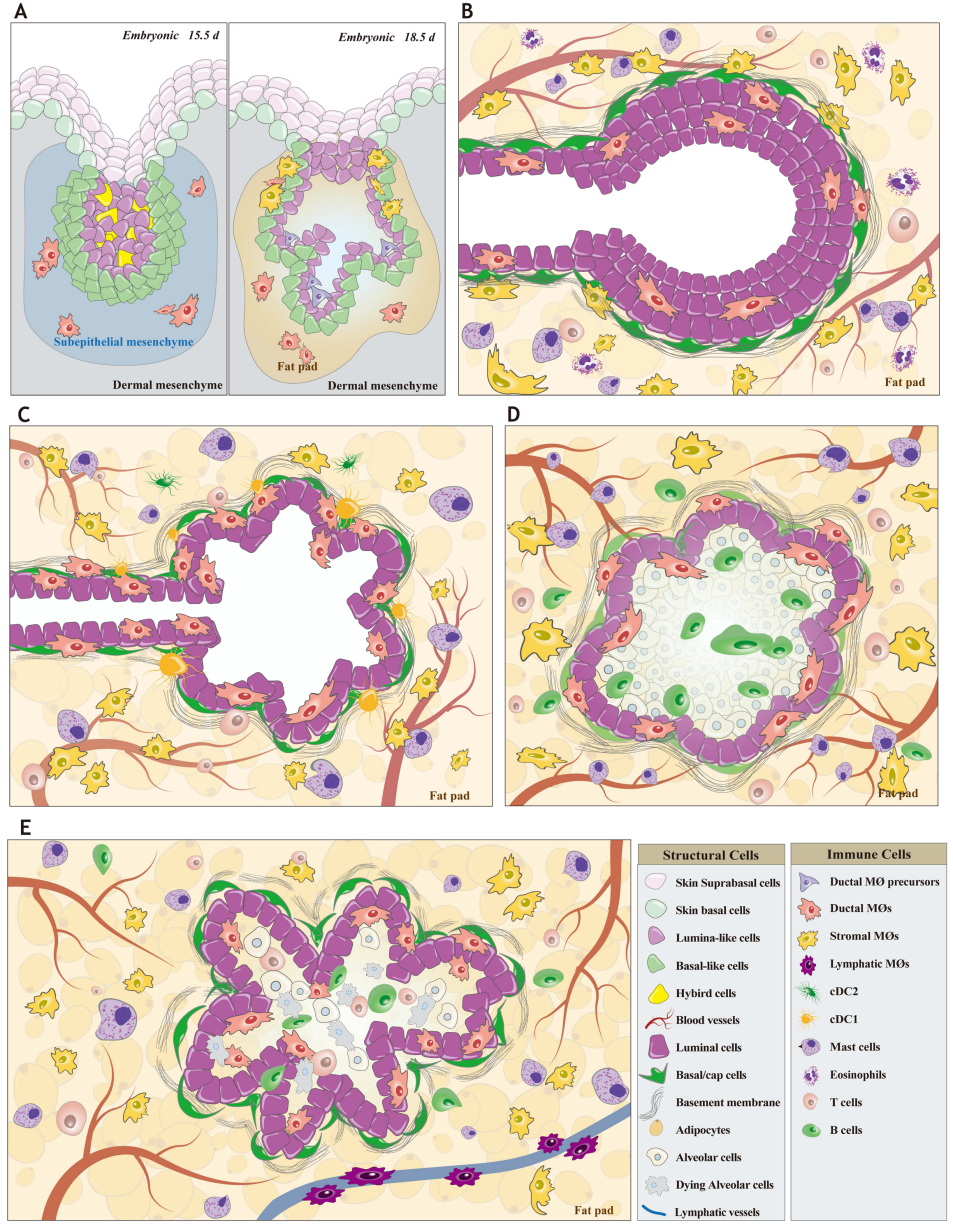
** Organization of the mouse mammary gland.** Diagram of the embryonic, puberty, pregnancy, lactation, and involution mammary in mice, depicting the various cell types, structures present, and their approximate location. **A) Embryonic stage:** The mammary bud forms and begins to invade the fat pad. At E15.5, a few ductal MΦs appear in the ductal region; by E18.5, ductal MΦs and their precursors emerge in the ducts, while stromal MΦs begin to populate the surrounding stroma. **B) Puberty:** Ducts elongate, branch, and form terminal end buds (TEB). Ductal MΦs reside within the ducts, while the surrounding stroma contains stromal MΦs, mast cells, T cells, and eosinophils. **C) Pregnancy:** Ductal branching continues and alveolar structures begin to form. The ductal and alveolar regions harbor ductal MΦs along with cDC1 and cDC2, whereas the stroma contains stromal MΦs, mast cells, and T cells. **D) Lactation:** Alveoli become fully differentiated and functionally active, lobules expand, and epithelial cells actively synthesize and secrete milk. Ductal MΦs and B cells are found in the alveolar region, while the stroma is predominantly populated by stromal MΦs, mast cells, and T cells. **E) Involution:** Alveoli collapse, epithelial cells undergo apoptosis, and the mammary gland gradually remodels back to a quiescent state. In the involuting alveoli, ductal **macrophages** and B cells are present; the stroma contains stromal MΦs, mast cells, and T cells; and lymphatic MΦs are distributed around the lymphatic vessels. (Created in Adobe Illustrator). **Abbreviations:** E, embryonic day; MΦ, Macrophage; TEB, terminal end bud; cDC, conventional dendritic cell.

Various immune cells, including macrophages, lymphocytes, eosinophils, neutrophils, mast cells, T cells, and dendritic cells, infiltrate the mammary gland at specific developmental stages and play pivotal roles in ductal growth, alveolar formation, milk secretion, and tissue remodeling, though their temporal dynamics are often described in broad stage-associated terms due to variable time windows across studies^52^-^56^. Accordingly, we have comprehensively reviewed mammary immune cell development across different stages (Figure 2).

### 3.1 Macrophages in the mammary gland: spatial distribution, heterogeneity, and functional roles

**Figure 3:**
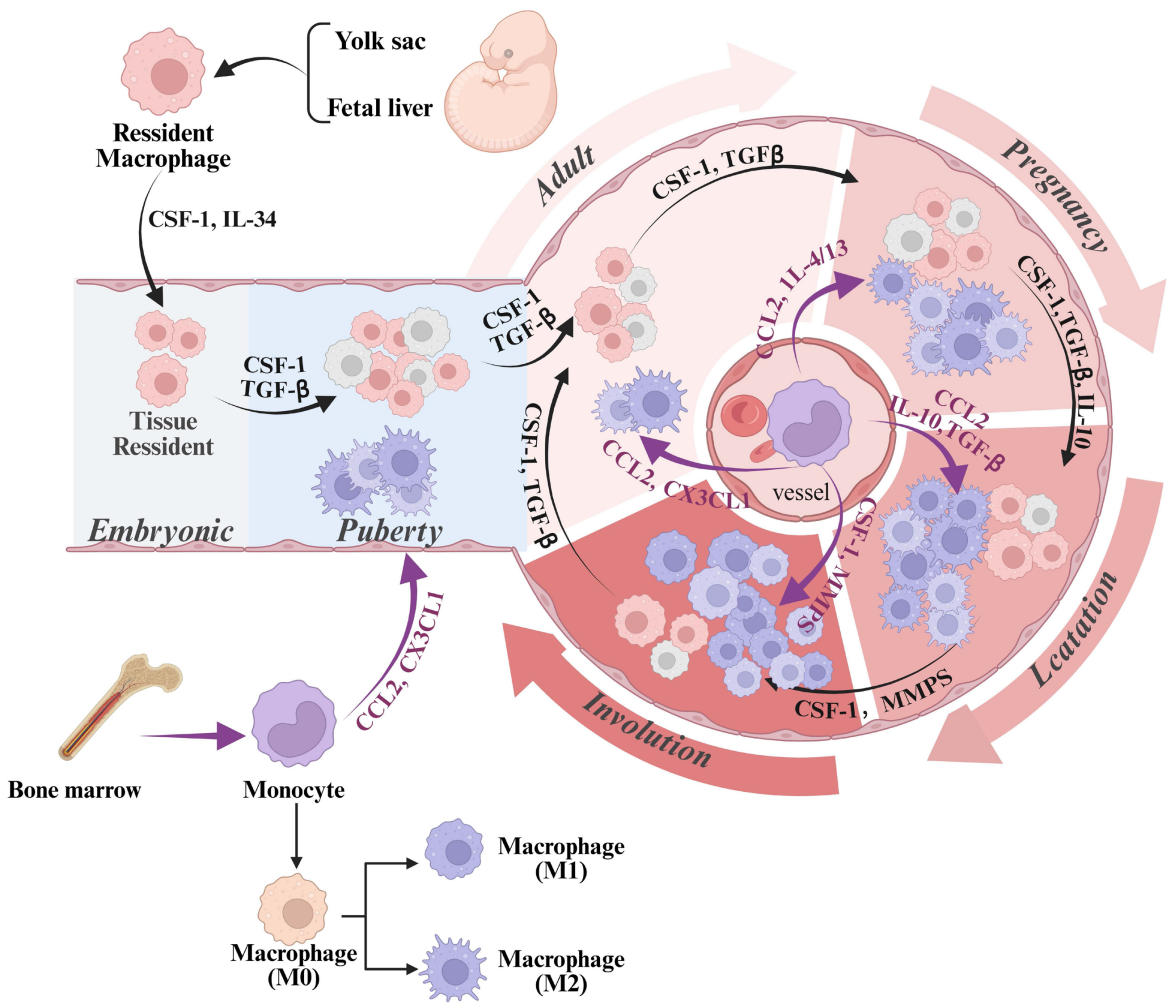
** Origin and development of mammary gland macrophages.** This schematic diagram illustrates the origin, differentiation, and stage-specific dynamics of mammary gland macrophages. Mammary macrophages originate from two lineages: embryonic precursors (yolk sac and fetal liver) and adult bone marrow-derived monocytes. During embryonic development and puberty, tissue-resident macrophages are maintained and expanded by factors such as CSF-1 and IL-34; at puberty, CSF-1 and CCL2 drive macrophage recruitment, contributing to stromal remodeling during ductal branching and terminal end bud formation. During pregnancy and lactation, cytokines including TGF-β, IL-4, IL-10, and IL-13 promote macrophage polarization toward an M2-like phenotype to support epithelial proliferation, alveologenesis, and milk secretion. During involution, CCL2, CSF-1, and CX3CL1 coordinate monocyte recruitment and macrophage polarization, with M1 macrophages participating in inflammatory responses and tissue clearance, while M2 macrophages promote tissue remodeling and repair. The diagram also depicts macrophage polarization from a resting state (M0) to pro-inflammatory (M1) and anti-inflammatory (M2) phenotypes. (Created in BioRender, https:/BioRender.com). **Abbreviations:** CSF-1, colony-stimulating factor-1; IL, interleukin; TGF-β, transforming growth factor-β; CCL2, chemokine ligand 2; CX3CL1, chemokine ligand 1.

Macrophages were first identified as key regulators of mammary gland development over two decades ago^56^. As the predominant immune cells in this process, they drive gland formation and growth. This section focuses on their development (Figure 3).

#### 3.1.1 Embryonic

Macrophages are innate immune cells originating from embryonic stem cells (derived from the fetal liver and yolk sac) as well as from circulating monocytes^57^, ^58^. During embryonic development, macrophages are present in the mammary gland. At embryonic day 14.5 (E14.5), their distribution differs between sexes: in male mice, macrophages contact and infiltrate the regressing mammary bud; in females, they reside mainly in the mesenchyme surrounding the epithelial bud^57^. However, the mechanisms and functions underlying this sexual dimorphism remain unclear. During this period, the mammary epithelium expands and branches, processes regulated by the Wnt/β-catenin and insulin-like growth factor 1 receptor (IGF-1R) pathways^59^. By E18.5, a substantial population of Cx3cr1^+^ macrophages appears in the stroma, particularly around the nipple, often near the epithelium. Cx3cr1 is a receptor for the chemokine CX3CL1 (C-X3-C motif chemokine ligand 1, also known as fractalkine), and this ligand-receptor pair mediates immune cell recruitment, adhesion, and functional regulation^60^. Additionally, unidentified MHCII^+^Cx3cr1^-^ immune cells have been observed near ductal tips^53^, ^61^. MHCII (major histocompatibility complex class II) molecules bind and present exogenous antigens to activate CD4^+^ T cells, initiating adaptive immunity^62^. By this stage, the mammary rudiments have matured into small ductal trees with 10-5 branches^63^. These findings suggest that embryonic mammary macrophages promote epithelial cell development and ductal branching, potentially through the expression of Wnt ligands^64^. Nevertheless, research on embryonic mammary macrophages remains limited, and their origin and regulatory mechanisms are largely unknown.

#### 3.1.2 Puberty

In contrast to pubertal events, macrophage development in the early postnatal mammary gland remains poorly understood. Although most macrophages are embryonically derived, bone marrow-derived monocytes may supplement this population during pubertal expansion^53^.

Pubertal mammary macrophages are classified by location into ductal and stromal subsets. Ductal macrophages (CD45^+^/F4/80^+^/CD11c^+^/CD11b^-^/MHCII^+^/Ly6C^-^/CD206^-^/Lyve1^-^/Cx3cr1^+^) reside between luminal and basal epithelial cells throughout the ductal network, with higher density near the nipple and lower density near alveoli^53^, ^61^, ^65^. They do not migrate but dynamically extend and retract dendrite-like to transiently contact one another and monitor the epithelium. Stromal macrophages (CD45^+^/F4/80^+^/CD11c^-^/CD11b^+^/CD206^+^/Lyve1^+^) are widely dispersed in the basal areas surrounding ducts^53^, ^65^. Fetal-derived CD206^Hi^ macrophages contact ductal structures and blood vessels, showing high capacity for scavenging blood-borne ligands^61^. Lyve1^+^ macrophages in the adipose stroma and fibrous capsule exhibit enhanced hyaluronan binding and degradation, contributing to extracellular matrix homeostasis in virgin mice^66^. However, the distinct embryonic origins of these subsets and the mechanisms governing their niche establishment remain unclear.

Recent studies have explored regulatory mechanisms of pubertal mammary macrophages. Brady et al. 67 showed that STAT5 in macrophages represses aromatase expression by binding to the *Cyp19a1* promoter. Loss of STAT5 increases aromatase and interleukin 6 (IL-6) expression, resulting in delayed ductal elongation, excessive branching, and epithelial proliferation. This ultimately accelerates ER-positive hyperplasia. Resident macrophages promote ductal morphogenesis and homeostasis via the tumor necrosis factor-α (TNF-α)-phosphatidylinositol 3-kinase (PI3K) -Cdk1/Cyclin B1 pathway, controlling epithelial proliferation and stem cell activity^68^. Macrophages also stimulate Wnt downstream of Notch to promote mammary stem cell function. They mediate Mcam-regulated development through Il4-Stat6-dependent Wnt5a secretion, activating non-canonical Wnt via Ryk in epithelial cells^69^. Mammary stem cell-derived Dll1 sustains macrophages and Wnt ligands, which feedback to reinforce stem cell stemness^70^. CCAAT/enhancer-binding protein beta (C/EBPβ) in macrophages facilitates alveolar budding during the luteal phase of the estrous cycle (diestrus in rodents) by modulating epithelial Wnt and Notch signaling, thereby linking systemic hormones to local stem cell expansion^52^. Collectively, these findings establish macrophages as central signaling hubs that integrate hormonal, inflammatory, and stem cell-derived inputs to coordinate mammary development. However, several key questions remain unanswered: what is the full repertoire of bidirectional signals beyond Dll1-Notch and Wnt signaling? How are these networks disrupted by oncogenesis? And what is the regulatory logic that enables STAT5 and C/EBPβ to direct stage-specific functions, such as ductal extension versus alveolar budding?

#### 3.1.3 Macrophage dynamics during mammary remodeling across reproductive stages

Throughout the life cycle of the mammary gland, the number, localization, and functional state of macrophages undergo precisely regulated changes that enable them to fulfil stage-specific roles in development, function, and remodeling.

During pregnancy, macrophages act as “architects” and “coordinators”. Positioned adjacent to expanding alveolar structures, they maintain close structural and functional relationships with the mammary epithelium^57^. The 40-fold increase in ductal macrophage numbers, which is disproportionate to epithelial expansion, suggests active recruitment and proliferation in response to pregnancy-specific signals rather than passive niche filling^53^. Macrophages facilitate alveogenesis by upregulating cell cycle and hormone-responsive genes to stimulate epithelial proliferation around terminal end buds, thereby supporting ductal elongation^53^, ^55^, and by activating STAT5 in epithelial cells to promote alveolar progenitor differentiation^67^. However, it is unclear whether this expansion results from the local proliferation of resident macrophages or the recruitment of circulating monocytes. Furthermore, the full range of paracrine and contact-dependent signals exchanged between macrophages and epithelial cells during alveogenesis remains to be discovered.

As the gland transitions to lactation, macrophage numbers stabilize and their functions adapt to support milk production. Residing near alveolar basal cells and within the peri-glandular stroma^57^, they diversify into distinct transcriptional subsets, including* Cd74*, *Socs3*,* Hsph1*, *Clec10a*, and *Birc5* expressing populations^65^. CD74^+^ macrophages exhibit a phagocytic phenotype, express MHC II-related genes and milk-protein transcripts, and clear epithelial debris. Socs3^+^ and Hsph1^+^ subsets contribute to immune surveillance via interferon gamma (IFN-γ), Toll-like receptor 4 (TLR4), and stress response pathways^65^. Lactation-induced macrophages (liMacs) arise via colony-stimulating factor 1 (CSF-1) -dependent but IL-34-independent mechanisms, with microbiota composition influencing their maturation^58^. Ductal macrophages maintain an immunosuppressive environment by increasing NF-κB, Notch, and EGF, and secreting anti-inflammatory mediators including IL-10 and Th17-related cytokines^53^. Although Birc5^+^ macrophages are associated with immune activation in disease^71^, their physiological role in lactation remains unknown.

During involution, macrophages acquire a phagocytic and tissue-remodeling phenotype, efficiently clearing cellular debris and restructuring the extracellular matrix. From day 4 post-weaning, they accumulate near regressing ducts and alveoli, with elevated numbers persisting until day 28, followed by resolution by 12 weeks^72^, ^73^. Their clearance function involves upregulating Mfge8 to bind phosphatidylserine on apoptotic cells and mediate efferocytosis^3^, while also phagocytosing residual milk lipids. CSF1R^+^ macrophages secrete proteases and growth factors that degrade the basement membrane and promote new matrix deposition^74^. Their immunomodulatory role evolves early pro-inflammatory signals initiate remodeling, followed by a shift toward an anti-inflammatory M2-like phenotype with TGF-β and IL-10 secretion^73^, ^74^.

In conclusion, the functional plasticity and heterogeneity of mammary gland macrophages are essential for reproductive success. However, key questions remain: What governs the precise spatiotemporal distribution of distinct macrophage subsets? What signals mediate their communication with other cells? And what upstream regulators orchestrate their sequential functional reprogramming across pregnancy, lactation, and involution?

### 3.2 Mast cells

#### 3.2.1 Origins of Mammary Mast Cells

Mast cells and macrophages share a developmental origin from yolk sac erythro-myeloid progenitors (EMPs). Immature mast cell progenitors appear in the circulation and peripheral tissues of mice and rats as early as E11.5^75^, ^76^, though their precise location within the embryonic mammary gland remains unknown. After birth, mast cells originate from bone marrow progenitors, enter the bloodstream, and spread to various tissues, where local environmental factors play a crucial role in shaping their final granule content and phenotype^56^, ^77^.

Resident mast cell establishment in the mammary gland involves two pathways: 1) the recruitment of progenitors to the mammary stroma followed by in situ maturation, and 2) direct migration of pre-formed, mature mast cells from neighboring subcutaneous connective tissue into the glandular parenchyma^77^. The homing of mast cell progenitors may be mediated by integrins. Integrin α4β7 is expressed by EMP-derived progenitors in the fetal liver at E11.5^75^ and by adult bone marrow progenitors with mast cell potential. It is essential for intestinal homing^78^. However, its specific role in mammary gland homing remains unclear. This integrin is also expressed by other immune lineage progenitors (e.g., T cells and innate lymphoid cells) and is generally considered a broad tissue-homing marker^79^. Therefore, it is unclear whether integrin α4β7 is specifically required for mast cell progenitor homing to the mammary gland. Furthermore, the homing of mast cells to the mammary gland under normal physiological conditions remains poorly characterized.

#### 3.2.2 Developmental Dynamics of Mast Cells in the Mammary Gland

Mast cell population dynamics within the mammary gland undergo distinct stage-specific changes. Before puberty, mast cells are present in small numbers without specific distribution. However, between postnatal weeks 5 and 8, their numbers increase significantly, with cells primarily residing near lymph nodes and blood vessels within the stromal regions of proliferating TEBs^77^. Following pregnancy, mast cells rapidly associate with expanding alveolar structures in the early stages^80^. Their population peaks at this initial stage (e.g., day 2 in rats) and then progressively declines throughout the remainder of pregnancy and into lactation^81^. After weaning and during involution, mast cells reappear rapidly to rebuild the adipose and fibrocellular stroma^82^.

#### 3.2.3 Mechanistic Roles in Tissue Remodeling Across Stages

This dynamic spatiotemporal localization enables mast cells to perform stage-specific remodeling functions. However, their role in pubertal ductal branching morphogenesis remains controversial. Early studies demonstrated that mast cell degranulation and serine protease activity promote proliferation and normal branching^77^. Nevertheless, this established model has been challenged by recent studies. Kapoor et al. 83 argue that prior conclusions relied heavily on the KitWsh mutant model. Using complementary genetic approaches, including a novel Ms4a2lsl-hDTR transgenic line, they found that neither constitutive nor pubertal-onset mast cell ablation impaired branching morphogenesis, calling into question an essential role for mast cells in this process^83^. This discrepancy is attributable to the KitWsh model itself, which harbors mutations affecting multiple cell lineages in addition to mast cells, thereby introducing potential confounding effects that cannot be attributed solely to mast cell deficiency.

Throughout the reproductive cycle, mast cells function as dynamic, stage-specific mediators of remodeling. During early pregnancy, they contribute to stromal angiogenesis by interacting with endothelial cells and stimulating pro-angiogenic factors such as angiogenin, endostatin, insulin-like growth factor-binding protein 3 (IGFBP-3), and monocyte chemoattractant protein 1 (MCP-1)^84^, ^85^. However, the factors limiting their involvement in vascular differentiation during late pregnancy and lactation remain unclear. The most defined role of mast cells emerges during involution. After weaning, falling prolactin induces gonadotropin-releasing hormone (GnRH) production and initial mast cell recruitment. GnRH produced by these cells then recruits additional mast cells, triggering massive tissue remodeling^86^. Mast cells drive epithelial apoptosis, adipocyte differentiation, and stromal remodeling through targeted release of active plasma kallikrein^87^.

In summary, mast cells play a critical role in mammary involution, coordinating apoptosis, matrix remodeling, adipocyte repopulation, and inflammation. However, the precise regulatory circuits controlling their recruitment and activation, the relative contributions of specific mediators, and their potential functional heterogeneity remain to be defined. Elucidating these mechanisms will clarify post-lactational remodeling and reveal new targets for remodeling-deficient disorders and postpartum breast diseases.

### 3.3 Lymphocytes

Although lymphocytes play a key role in adaptive immunity and have been identified in the mammary gland from pre-puberty through adolescence^88^, most studies have focused on their recruitment and functions during the reproductive cycle, particularly lactation. This section reviews their spatiotemporal distribution across developmental stages and discusses their emerging roles in mammary gland remodeling.

#### 3.3.1 T cell development and remodeling functions in mammary tissue

T cells originate from bone marrow progenitors that migrate to the thymus for maturation before seeding peripheral tissues^89^. Although their developmental origin is well established, the mechanisms guiding their specific homing to the mammary gland remain under investigation. Two distinct migration pathways have been proposed. One well-supported route suggests a gut-mammary axis, whereby T cells migrate from the intestine to the mammary gland during late gestation in a microbiota-dependent manner^90^, ^91^. In contrast, a recent study revealed a more direct pathway, showing that a specific subset of thymus-derived intraepithelial lymphocyte precursors preferentially colonizes the mammary gland over the gut during pregnancy^92^.

T cells are pivotal regulators of mammary gland biology, contributing to tissue remodeling, mucosal barrier integrity, and immune homeostasis throughout postnatal development. Their distribution and functional states evolve across stages to meet changing physiological demands. During puberty, CD4⁺ and CD8⁺ T cells accumulate around growing ducts in mice, suggesting a role in ductal morphogenesis^54^. During late pregnancy, T cells support the functional maturation of alveolar epithelium, facilitating the transition to lactation^91^, ^92^.

Lactation is marked by significant expansion in T cell diversity and abundance, including CD4⁺ and CD8αβ⁺ effector T cells, CD8αα⁺ T cells, and innate-like γδ T cells 90, 93. These subsets occupy distinct niches: in bovine mammary tissue, CD4⁺ T cells localize between connective tissue and lobules, CD8⁺ T cells reside near alveolar epithelium^94^. T cell recruitment during lactation may involve mucosal homing pathways. Expression of mucosal addressin cell adhesion molecule 1 (MAdCAM-1) is upregulated during pregnancy and correlates with T cell numbers, supporting an "entero-mammary axis" similar to that described for IgA⁺ plasma cells^95^. Functionally, these T cells shape the local immune milieu by secreting T cell-polarizing cytokines: undifferentiated cells produce Th1-type cytokines, while differentiated cells secrete Th2-type cytokines^96^. IFN-γ-producing CD4⁺ Th1 cells engage with antigen-presenting cells to fine-tune epithelial reorganization and luminal lineage differentiation during postnatal organogenesis^54^. Additionally, many mammary lymphocytes are released into human milk, where T cells constitute up to 83% of lymphocytes^97^. Among these, CD8⁺ T cells predominate (75%) and can migrate to neonatal Peyer's patches, potentially supporting infant immune development^98^.

During involution, the T cell compartment shifts toward immunoregulatory subsets, including expansion of CD2⁺ T cells and RORγt⁺ CD4⁺ regulatory T cells (Tregs)^99^. Accordingly, CD2⁺ T cells constitute 80-90% of lymphocytes in bovine involuting milk, declining to 50-60% in mature milk^100^. This period is also characterized by tolerogenic dendritic cells, which suppress effector T cell responses and promote Treg-mediated tissue homeostasis^99^.

#### 3.3.2 B cell development and remodeling functions in mammary tissue

Research on B lymphocytes in the mammary gland remains limited. In lactating sows, IgA⁺ B cells are predominantly localized in the basal region of the mammary gland^101^, though their distribution in other species is not well characterized. The origin of mammary B cells follows a well-established entero-mammary pathway. B cells are initially activated by antigens in gut-associated lymphoid tissue, particularly in Peyer's patches of the small intestine, which serve as the primary source of IgA⁺ plasma cells for the mammary gland^102^. These activated B cells then migrate through the bloodstream to mammary tissue, guided by the chemokine C-C motif chemokine ligand 28 (CCL28), where they differentiate into IgA-secreting plasma cells and are subsequently excreted into milk^95^.

While the role of mammary-derived IgA in providing passive antibacterial immunity to suckling neonates is well documented^103^, the mechanistic contributions of B-lineage cells to mammary gland morphogenesis and tissue remodeling remain poorly understood. It is unclear how distinct B cell subsets are recruited to and retained within specific mammary niches during development, or what molecular signals drive their local adaptation. Furthermore, whether B cells directly shape the tissue microenvironment through antigen presentation or cytokine secretion remains an open mechanistic question.

### 3.4 Other immune cells in the mammary tissue play a role in remodeling function

#### 3.4.1 Eosinophils

Eosinophils originate from bone marrow progenitors, and their proliferation, differentiation, recruitment and activation are regulated by IL-5, granulocyte-macrophage colony-stimulating factor (GM-CSF), and IL-3^104^, ^105^. They migrate along chemokine gradients via CCR3 binding^106^, which is primarily induced by CCL11/eotaxin-1 and is partly dependent on IL-5^107^. During puberty, eosinophils are recruited to TEBs, where they collaborate with macrophages to promote ductal branching^56^. IL-5-deficient mice exhibit reduced TEB numbers, diminished branching complexity, and decreased overall mammary gland density^108^. Conversely, IL-5 overexpression leads to sustained eosinophil accumulation and has been associated with delayed mammary development during puberty and impaired TEB formation^109^. These developmental delays are transient, however, and are resolved at later stages, suggesting that eosinophils possess regulatory thresholds that govern TEB development. The precise mechanisms involved remain undefined.

The precise localization of eosinophils within the mammary gland during pregnancy, lactation, and involution, along with their associated functions and extracellular matrix interactions, has not been comprehensively documented. Some studies suggest that eosinophil populations begin to increase from day 4 of involution^72^, but their role in mammary regression remains unknown.

#### 3.4.2 Neutrophils

Neutrophils play a key role in clearing pathogens through processes, such as phagocytosis, degranulation, and neutrophil extracellular trap (NET) formation 110. However, their involvement in mammary gland development remains largely unexplored. During pregnancy, acinar cells comprise ~3% of mammary immune cells^65^. During development, neutrophil transmigration across the blood-milk barrier depends on the integrin CD11b/CD18 111.

Neutrophil recruitment depends on macrophage-derived TNFα, IL-1β, and IL-8 112. Furthermore, an elevated neutrophil count is a predictive marker for mastitis and functions as a diagnostic indicator for breast infections in ruminant milk production systems^113^. Under physiological conditions, there are few neutrophils, but they are rapidly recruited upon infection^114^, although they may also cause tissue damage^112^. The mechanisms governing neutrophil functions and cellular interactions across stages warrant further study.

Although the distribution and roles of immune cells in mammary remodeling are well documented, the mechanisms by which they communicate with each other remain unclear. During infection, epithelial cells recruit immune cells via cytokines (IL-6, IL-1β and TNF-α) and chemokines such as C-C motif chemokine ligand 2 (CCL2), C-X-C motif chemokine ligand 1 (CXCL1), and CCL28^115^-^117^. However, the mechanisms of recruitment during normal development remain unclear.

## 4. Dynamic changes in mammary gland amino acids and transporters

Different metabolic demands across mammary developmental stages drive stage-specific fluctuations in amino acids. During late pregnancy, amino acid deposition increases to support mammary growth^118^, ^119^. During lactation, amino acid profiles undergo species-specific alterations^120^. Given limited studies on involution, this section focuses on puberty, pregnancy, and lactation, with stage-dependent changes in blood and milk amino acids summarized in the following sections.

### 4.1. Characteristics of free amino acids in blood

Tables 1-3 summarize the changes in blood amino acid levels during puberty, pregnancy, and lactation in mice^121^-^127^, dairy cows^128^-^135^, and pigs^136^-^148^. Most free amino acids in these species exhibit stage-dependent fluctuations that parallel the metabolic demands of growth and reproduction. Concentration generally rises from puberty to adulthood, peaks during gestation, and partially recovers after weaning. However, the pattern during lactation differs markedly across species: in pigs, most amino acids decline upon transitioning to lactation. In contrast, in mice and cows, several amino acids, including glutamate, glutamine, and alanine, increase during this period, indicating species-specific metabolic strategies.

### 4.2 Characteristics of free amino acids in milk

Milk amino acid levels across species during lactation are shown in Table 4,^127^, ^137^, ^149^-^157^. The concentrations of free amino acids in milk differ between colostrum and mature milk, with glutamic acid being the most abundant across species. Other core dominant amino acids include branched-chain amino acids, arginine, and glutamine. Notably, there are species-specific differences in the direction and magnitude of changes from colostrum to mature milk. Currently, there is a lack of longitudinal paired data on blood and milk amino acids from the same individuals across different physiological stages. Consequently, there is a significant research gap regarding amino acid secretion patterns during non-lactating stages.

### 4.3 Characteristics of amino acid transporters in the mammary gland

**Figure 4:**
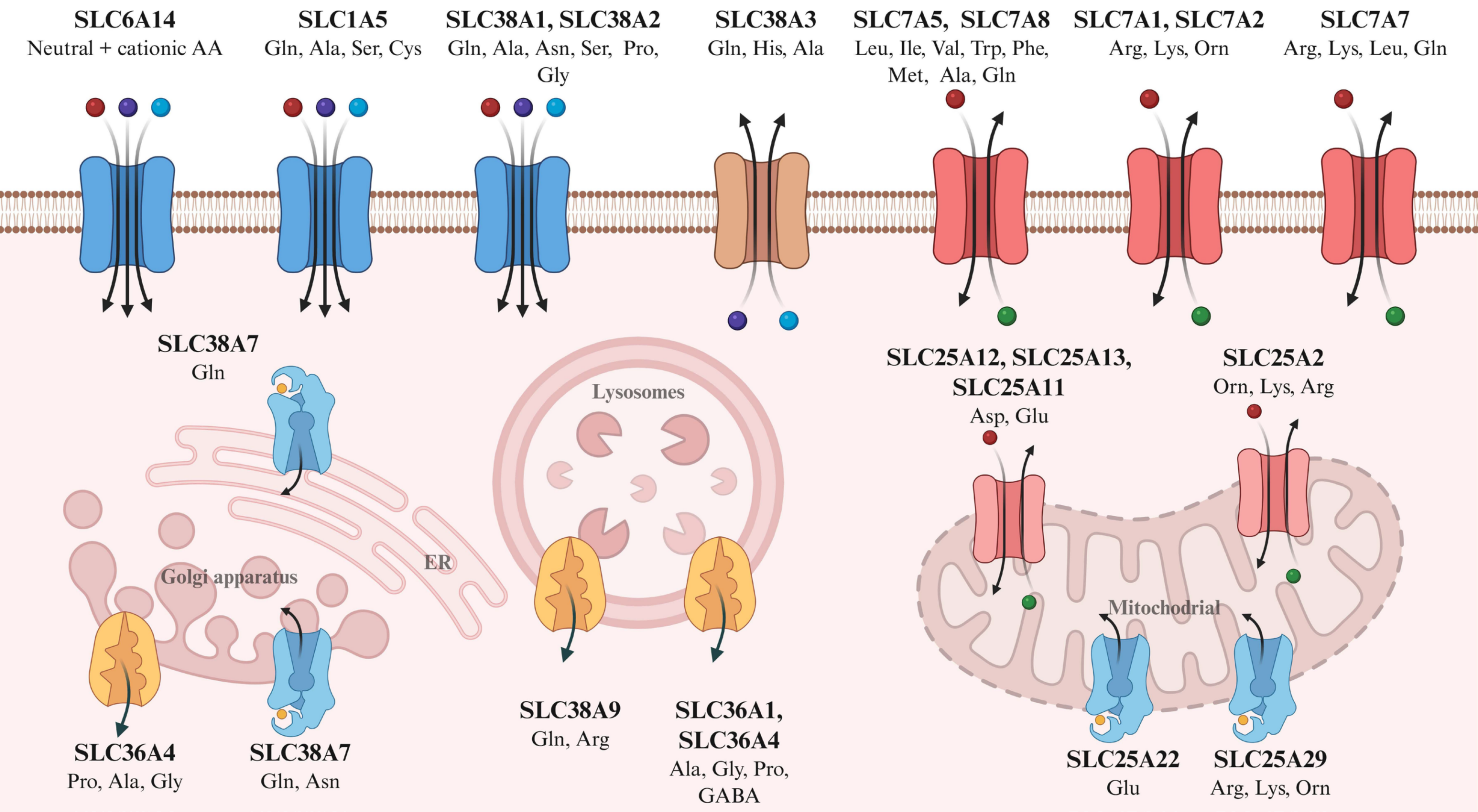
** Subcellular localization of amino acid transporters in mammary epithelial cells.** Schematic diagram showing the distribution of amino acid transporters across major subcellular compartments (plasma membrane, lysosome, mitochondrion, Golgi apparatus, and endoplasmic reticulum) in mammary epithelial cells. **Plasma membrane:** sodium-dependent transporters (SLC1A5, SLC38A1/2/3, SLC6A14) mediate concentrative amino acid uptake, while sodium-independent transporters (SLC7A5/7A8, SLC7A1/7A2, SLC7A7) mediate amino acid exchange. **Lysosome:** SLC38A9 and SLC36A1 mediate amino acid efflux from the lumen to the cytosol. **Mitochondria:** SLC25 family carriers (SLC25A12/13/11/22/29) regulate amino acid exchange across the inner membrane. **Golgi apparatus** and** endoplasmic reticulum,** SLC38A7 and SLC36A4 are involved in amino acid transport into secretory vesicles. Substrate specificities for each transporter are indicated in the diagram. (Created in BioRender, https:/BioRender.com). **Abbreviations:** AA, amino acid; BCAA, branched-chain amino acid; Gln, glutamine; Arg, arginine; Leu, leucine; Ile, isoleucine; Val, valine; Ala, alanine; Pro, proline; Gly, glycine; Asp, aspartate; Glu, glutamate; GABA, gamma-aminobutyric acid.

Mammary amino acid transporters fall into two categories: sodium-dependent cotransporters (*SLC1A5*, *SLC38A1/2/3*, and* SLC6A14*) for concentrative uptake, and sodium-independent exchangers (*SLC7A5/7A8*, *SLC7A1/7A2*, and* SLC7A7)* for homeostasis^158^. The mammary gland also expresses peptide transporters PEPT1/2 for di-/tripeptide uptake^159^ and lysosomal proton-coupled transporters (*SLC36A1* and *SLC36A4*) for amino acid efflux^160^. The subcellular localization of these transporters in mammary epithelial cells is illustrated in Figure 4.

Their expression is stage-specific: *SLC1A5* is upregulated during early pregnancy and again at the peak of lactation, while *SLC7A5*/*SLC7A8* and *SLC7A1* are only induced during lactation. Conversely, excitatory amino acid carrier 1 (EAAC1), glutamate aspartate transporter (GLAST), and the taurine transporter decline from pregnancy through to lactation^161^. This pattern suggests regulation by lactogenic hormones: insulin enhances the expression of *SLC7A5* and the uptake of lysine and arginine^162^, while prolactin upregulates the localization of *SLC7A5*^163^. Despite our understanding of their developmental and hormonal regulation, the potential role of mammary amino acid transporters in integrating nutrient supply with immune cell metabolism and function requires further investigation.

## 5. Amino acid metabolism in the mammary gland and its immunomodulatory mechanisms

**Figure 5:**
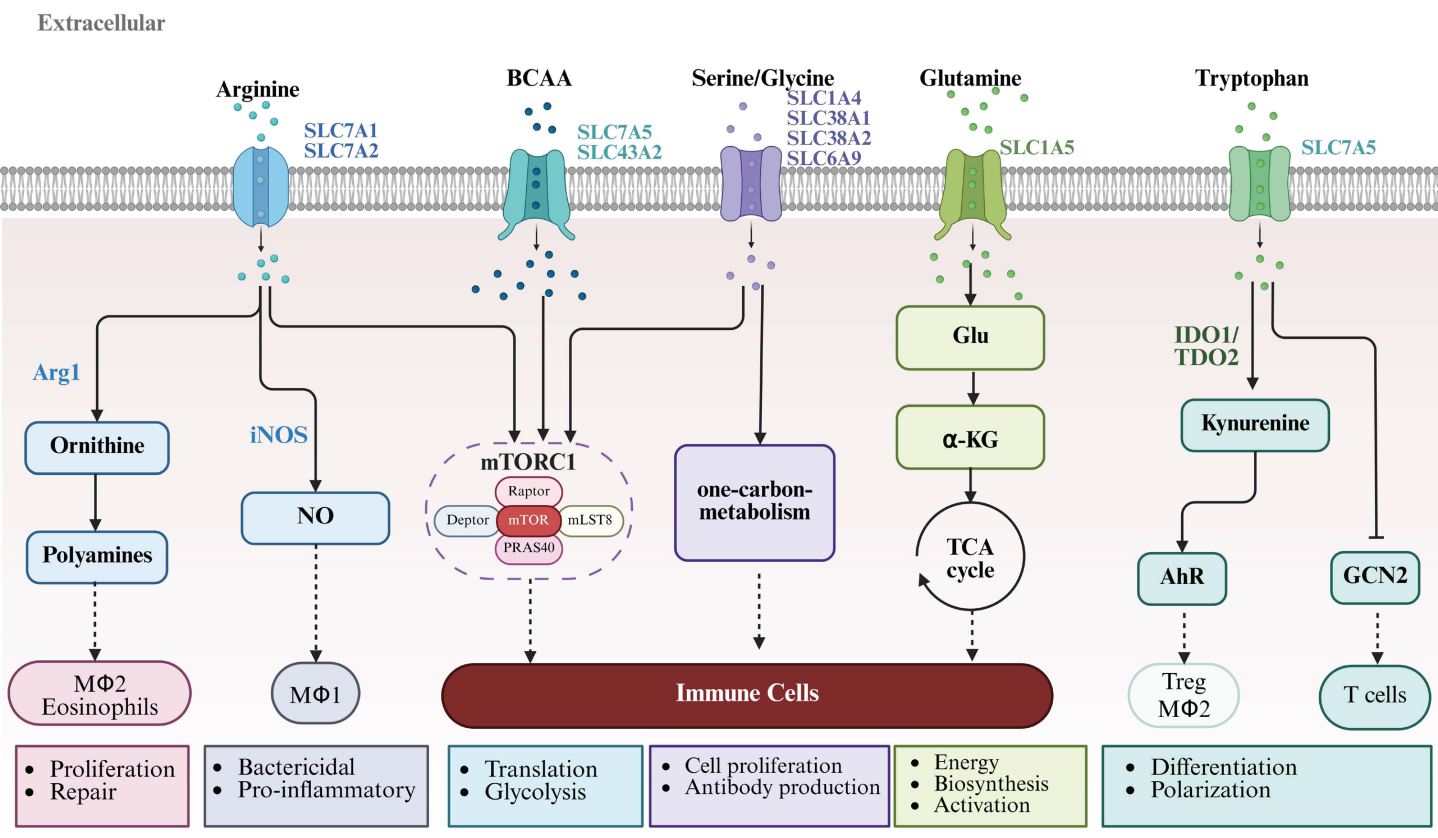
** Amino acid metabolism regulates immune cell function and polarization through distinct metabolic pathways.** This schematic diagram illustrates how different amino acids (arginine, branched-chain amino acids, serine/glycine, glutamine, and tryptophan) enter immune cells via specific transporters and subsequently modulate immune cell functional polarization and metabolic reprogramming through distinct downstream pathways. Arginine, imported via* SLC7A1* and* SLC7A2*, is metabolized by Arg1 to produce ornithine and polyamines, promoting tissue repair and proliferation mediated by M2 macrophages and eosinophils; alternatively, arginine is metabolized by inducible iNOS to generate NO, driving pro-inflammatory responses and glycolytic reprogramming in M1 macrophages. BCAAs, glutamine, and serine/glycine are taken up through transporters such as *SLC7A5*, *SLC1A5*, and *SLC38A1/2*, then enter one-carbon metabolism and the tricarboxylic acid (TCA) cycle, providing energy and biosynthetic precursors while activating the mTORC1 signaling pathway to support immune cell proliferation, differentiation, and antibody production. Tryptophan, imported via *SLC7A5,* is metabolized by IDO1/TDO2 to produce kynurenine, which activates AhR and promotes the polarization of Tregs and M2 macrophages. In addition, tryptophan deprivation can regulate T cells activation and tolerance through the GCN2 pathway. The diagram also highlights the central role of the mTORC1 complex (comprising mTOR, Raptor, mLST8, and PRAS40) in integrating amino acid signals and regulating immune cell metabolism and function. (Created in BioRender, https://BioRender.com). **Abbreviations:** BCAA, branched-chain amino acid; Arg1, arginase 1; iNOS, inducible nitric oxide synthase; NO, nitric oxide; IDO1, indoleamine 2,3-dioxygenase 1; TDO2, tryptophan 2,3-dioxygenase; AhR, aryl hydrocarbon receptor; Treg, regulatory T cell; GCN2, general control nonderepressible 2 kinase; mTORC1, mechanistic target of rapamycin complex 1; TCA, tricarboxylic acid; α-KG, alpha-ketoglutarate.

Amino acids are essential for mammary gland development, with each physiological stage exhibiting a distinct metabolic profile. As well as serving as protein precursors, amino acids act as dynamic signaling molecules that orchestrate the proliferation, differentiation, functional polarization, and metabolic reprogramming of immune cells within the mammary tissue microenvironment (Figure 5). Due to limited data on other stages, this section focuses on amino acid metabolism during lactation and its immunomodulatory roles in the mammary gland.

### 5.1 Glutamine

During the first six months of human lactation, free glutamine and glutamate increased by up to 35% and 40% in milk, respectively^164^. In lactating sows, milk glutamine output exceeds its uptake from blood^44^, indicating that the mammary gland must rely on local synthesis to meet the high demand for glutamine. However, the porcine mammary gland lacks two key enzymes required to convert arginine, ornithine, or proline into glutamine^165^; instead, it utilizes BCAAs as precursors for glutamine synthesis via α-ketoglutarate^44^. This pathway is constrained by α-ketoglutarate availability and glutamine synthetase (GS) activity. In mouse models, GS is confined to adipocytes and is lower during lactation than in the non-lactating state^166^. Thus, whether modulating GS expression or α-ketoglutarate availability can promote mammary development via enhanced glutamine synthesis is still unclear.

Glutamine serves as a critical metabolic substrate for immune cells and plays a pivotal role in the execution of immune defense functions by immune cells within the lactating mammary gland^167^. Previous studies in dairy cows have demonstrated that glutamine supplementation increases CD4⁺ T cell populations and elevates levels of associated cytokines, including IL-1β, IL-6, and IL-10^168^, ^169^. Consistent with this, activated T cells strongly upregulate the expression of key glutamine transporters, including* SLC38A1* (SNAT1), *SLC38A2* (SNAT2), and *SLC1A5* (ASCT2), thereby ensuring sufficient glutamine availability to fuel their metabolic needs^170^, ^171^. Our previous research demonstrated that glutamine promotes intestinal sIgA secretion via the microbiota and IL-13^172^, while subsequent studies further revealed that the intestinal immunoprotected effects of glutamine are attributable to microbial metabolites rather than the microbiota itself^173^. However, whether the regulatory effects of glutamine on mammary-resident immune cells during lactation are attributable to similar or distinct mechanisms remains an open question.

### 5.2 Arginine

Arginine is metabolized through two primary pathways: polyamine synthesis (critical for cell growth and differentiation) and nitric oxide (NO) generation (a key signaling molecule in immune and metabolic regulation). Within mitochondria, arginine is catabolized by arginase 1/2 to form ornithine, proline, and urea^174^. These metabolites can promote mammary gland development and stimulate the proliferation of mammary epithelial cells and lipogenesis, potentially via mechanisms involving the mTOR pathway and miRNA regulation^175^, ^176^. In lactating pigs, the mammary gland contains highly active arginase II and ornithine aminotransferase in the mitochondria, as well as highly active arginase I in the cytoplasm, providing the conditions for efficient arginine utilization^165^. Notably, there is a dynamic interaction between arginine metabolism and the mammary microbiome: arginine metabolites can influence the composition of the microbiome, which in turn affects local arginine absorption and metabolism^177^. Thus, although the mammary gland exhibits a robust capacity for arginine metabolism and dynamic interaction with the local microbiome, the manner in which these factors coordinate to support mammary development remains unclear.

Previous studies in intestinal models have demonstrated that arginine modulates immune responses through cytokines and the intestinal microbiota^178^, which suggests that similar regulatory mechanisms may be at work in the mammary gland. Arginine-derived polyamines support the proliferation and function of various immunosuppressive cell types, including myeloid-derived suppressor cells, macrophages, and regulatory T cells. In contrast, NO drives glycolytic reprogramming and reinforces the pro-inflammatory phenotype of M1 macrophages^179^. During mastitis, arginine supplementation increases NO levels in mammary tissue, thereby enhancing macrophage antibacterial capacity and stimulating epithelial proliferation to accelerate tissue repair^180^. During the tissue repair phase of involution, metabolic flux switches from inducible nitric oxide synthase (iNOS) - to Arg1-dominant arginine catabolism, leading to ornithine and polyamine accumulation, which promotes M2 macrophage polarization and supports immune-mediated tissue remodeling^179^. It has been proposed that arginine metabolism orchestrates immune cell polarization and function by supplying key metabolites that shape the inflammatory or reparative microenvironment. However, it is unclear whether arginine directly modulates the function of mammary-resident immune cells during different physiological stages, highlighting the need for further research.

### 5.3 Tryptophan

Tryptophan is metabolized primarily through three pathways (kynurenine, 5-hydroxytryptamine, and indole pathways)^181^, with the kynurenine pathway serving as the central catabolic route and a critical hub for immunoregulation. This pathway is initiated by tryptophan 2,3-dioxygenase (TDO) and indoleamine 2,3-dioxygenase 1/2 (IDO1/2), which convert tryptophan into kynurenine and subsequently generate a series of downstream metabolites^182^.

Tryptophan depletion alone has significant effects on immune cell function since T cells, particularly effector T cells, are highly sensitive to tryptophan availability^183^. Local tryptophan depletion activates the general control nonderepressible 2 (GCN2) kinase pathway, which promotes Treg differentiation and maintains immune tolerance by restraining excessive inflammation^184^. This pathway also inhibits mTORC1 and promotes oxidative phosphorylation, further reinforcing Treg differentiation^185^. Tryptophan metabolism plays a critical role in maintaining immune tolerance within the lactating mammary gland. Recent studies indicate that the tryptophan metabolite 5-hydroxyindoleacetic acid (5-HIAA) mitigates vagotomy-induced mastitis, a protective effect associated with AhR activation and subsequent suppression of the NF-κB pathway^45^. AhR signaling promotes a tolerogenic phenotype in dendritic cells, suppresses Th17 cell differentiation, and enhances Treg generation, collectively restraining excessive inflammation^186^. However, the specific mechanisms by which tryptophan and its metabolites contribute to immune homeostasis in the healthy mammary gland under physiological conditions remain unexplored.

### 5.4 BCAAs

BCAAs are extensively metabolized in the mammary gland during lactation^44^. Once inside mammary epithelial cells, BCAA catabolism is initiated by branched-chain aminotransferase (BCAT), including the mitochondrial isoform BCATm and the cytosolic isoform BCATc, generating branched-chain ketoacids (BCKAs) and glutamate^44^, ^187^. The activity of key catabolic enzymes is dynamically upregulated during lactation. In non-lactating rats, only 20% of branched-chain ketoacid dehydrogenase (BCKAD) is active in the mammary gland^188^. Throughout lactation, BCKAD remains fully active, and BCAT activity increases tenfold^189^. Our previous study demonstrated that increasing the valine-to-lysine ratio in low-protein lactating sow diets enhances BCAA transport and catabolism in the mammary gland and piglet jejunum^190^.

BCAAs regulate metabolic reprogramming of immune cells through nutrient sensing pathways. Our studies have demonstrated that leucine supplementation improves intestinal health via sIgA secretion^191^, that valine supplementation during late gestation promotes mammary gland development in gilts^192^, and more recently that valine supplementation at an optimal SID Val/Lys ratio of 0.88 in low-protein lactating sow diets enhances mammary sIgA production and intestinal immune maturation in suckling piglets^193^. At the immune cell level, BCAAs fine-tune immune responses by modulating mTOR-dependent lymphocyte proliferation and metabolic programming^194^. Specifically, BCAA accumulation promotes the effector function and antitumor immunity of CD8⁺ T cells by reprogramming glucose metabolism^195^. Moreover, isoleucine maintains the proliferative state of Treg cells through* SLC3A2*-dependent metabolic reprogramming^196^. The *SLC7A5*-*SLC3A2* transporters further activate mTORC1 to promote T cell metabolic reprogramming^197^, ^198^. Activated B cells also depend on BCAAs, exhibiting a significant increase in amino acid uptake mediated by *SLC7A5* and *SLC1A5,* along with the stabilizing chain *SLC3A2*^199^. For macrophages, BCAA supplementation promotes M2 polarization both in vitro and in vivo and increases oxidative phosphorylation in M2 macrophages^200^. Thus, although BCAAs influence immune cell function through metabolic reprogramming, whether they direct immune cell recruitment and functional polarization in the mammary gland across different developmental stages remains an open question.

## 6. Amino acid metabolisms in mammary intercellular communication

Amino acid metabolism facilitates critical communication between immune cells and mammary epithelial cells, coordinating tissue development and function. This crosstalk occurs via metabolite exchange, cytokine signaling, and extracellular vesicle transmission. For example, epithelial-derived glutamine can fuel adjacent macrophages, while macrophages may supply arginine and polyamines to support epithelial proliferation^177^.

Cytokine signaling is another key mediator. IFN-γ upregulates IDO expression, enhancing tryptophan catabolism to produce immunomodulatory kynurenine^184^. Conversely, IL-4 and IL-13 upregulate Arg1, shifting arginine metabolism toward polyamine production^179^. These cytokines thus practically regulate the metabolism and function of adjacent cells.

Extracellular vesicles, such as exosomes, participate in the process by transporting metabolic enzymes and metabolites between cells. Within the mammary microenvironment, for example, immune and epithelial cells use exosomes to exchange metabolic information and coordinate tissue-level function^177^. This intercellular communication network adapts dynamically across developmental stages. During lactation, metabolic crosstalk between immune and epithelial cells coordinates milk synthesis with immune defense^167^. During involution, similar crosstalk facilitates the coordinated tissue clearance and remodeling^201^. Therefore, amino acid metabolism underpins a critical communication system that ensures the orderly progression of mammary gland development and function^177^.

## 7. Conclusion

Overall, the mammary gland is a unique physiological model for dissecting immunometabolism crosstalk in postnatal tissue development, dynamic remodeling, and functional homeostasis. Substantial studies have confirmed that stage-specific reprogramming of amino acid metabolism matches the dynamic physiological demands across key mammary developmental windows, and mammary tissue-resident immune cells (predominantly macrophages) are well-documented core regulators of mammary morphogenesis, alveolar maturation, and tissue remodeling. Together, they form a core metabolic-immune axis driving mammary tissue plasticity and functional adaptation.

However, critical knowledge gaps remain unaddressed. Most existing work focuses on pregnancy and lactation, while amino acid metabolic dynamics and their crosstalk with resident immune cells during embryogenesis, puberty and involution are poorly characterized. The functions of less-studied immune subsets and the controversial role of mast cells in pubertal ductal morphogenesis require further validation, and most current evidence is associative, lacking causal verification via functional studies. For livestock production, mammary function directly determines lactation performance and economic benefits. Future systematic studies on this metabolic-immune axis will advance fundamental understanding of mammary biology and provide novel nutritional and immunological targets to improve livestock lactation performance and prevent mammary diseases.

## Figures and Tables

**Table 1:** Plasma free amino acid concentrations across different physiological stages in mice.

AA (μM)	Puberty	Adult	Gestation	Lactation	Post-weaning
Ile	87.90	140.50	96.80	118.40	143.70
His	49.65	92.50	20.00	70.50	-
Met	31.95	63.50	164.75	271.00	160.30
Val	175.25	264.50	210.45	342.95	399.70
Lys	253.75	443.00	554.40	421.95	437.20
Leu	136.60	203.50	126.55	201.50	229.70
Trp	70.80	194.50	101.95	80.35	150.90
Phe	56.65	63.00	29.00	102.00	-
Thr	117.15	159.50	556.15	271.20	245.20
Arg	69.55	124.50	127.20	168.90	120.80
Gly	195.15	263.50	104.65	125.90	144.20
Ser	110.00	127.50	116.85	107.95	150.60
Tyr	51.45	122.50	30.00	86.70	-
Glu	19.35	38.50	61.95	354.00	35.70
Gln	378.55	811.00	592.05	312.70	508.00
Ala	257.45	631.00	523.00	320.00	-
Pro	40.70	99.00	143.00	149.00	-
Asp	21.35	30.00	17.75	14.45	10.50
Asn	21.65	45.50	23.00	56.90	-
Cys	48.40	11.00	51.80	34.80	45.50
Tau	411.00	464.50	810.00	220.00	-
Cit	43.00	89.50	37.70	46.10	87.40
Orn	57.00	75.50	60.95	80.25	69.50

Puberty (PND 21-42): total n=13; normal mice (n=5)[121] and A/J mice (n=8)[122]. Adult (PND 70-98): total n=5; NRG mice(n=2)[123] and normal mice (n=3)[124]. Gestation (GD 0.5-14): total n=22; C57BL/6J mice(n=12)[125] and normal mice (n=10)[126]. Lactation (LD 14-20): total n=20; normal mice (n=10)[126] and ICR mice (n=10)[127]. Post-weaning (PWD 2): normal mice (n=10)[126]. “-” indicates that the value was not reported in the original source. All values are averages of data from published literature. PND: postnatal day; GD: gestational day; LD: lactation day; PWD: Post-weaning day.

**Table 2:** Plasma free amino acid concentrations across different physiological stages in dairy cows.

AA(μM)	Nursery	Lactation					Dry period^a^	
		1W	2W	3W	4W+		2W pre	4W pre
Ile	24.65	87.35	108.10	113.70	101.00		126.15	98.25
His	7.09	41.20	46.10	44.90	61.00		55.50	39.90
Met	4.95	23.65	24.70	24.40	14.80		24.30	12.92
Val	62.61	184.80	228.80	235.40	257.00		262.35	200.50
Lys	34.03	48.65	61.40	60.70	64.30		76.55	55.60
Leu	41.27	140.89	165.04	161.29	155.48		155.05	145.94
Trp	-	31.46	40.99	42.84	46.50		36.39	35.33
Phe	13.01	50.62	52.46	46.90	50.20		50.96	45.81
Thr	32.61	67.85	97.40	105.00	153.00		87.85	65.90
Arg	10.20	41.10	50.40	54.90	54.20		72.55	52.25
Gly	36.99	428.02	499.35	466.11	415.55		233.33	219.81
Ser	38.88	102.12	113.71	115.82	95.56		87.96	73.74
Tyr	12.03	34.78	41.63	47.05	55.41		49.11	46.40
Glu	4.81	78.22	77.54	84.14	112.11		77.91	80.99
Gln	44.85	261.20	259.00	254.20	-		304.05	-
Ala	94.44	220.52	211.38	237.16	216.97		214.98	201.43
Pro	27.27	73.01	80.64	87.65	90.86		71.09	62.85
Asp	1.02	5.02	6.55	7.84	11.33		6.56	7.50
Asn	3.07	27.10	41.30	42.40	-		30.00	26.10
Cys	-	74.07	71.35	77.53	55.34		72.41	71.60
Tau	1.73	-	-	-	-		25.90	28.45
Cit	-	-	-	-	-		80.00	54.20
Orn	0.49	-	-	-	-		55.10	26.15

^a^ Dry period: 60 d prior to second calving, corresponding to GD 220-280 of second gestation. Nursery (PND 0-90): total n=52 (n=46[128], 6[129]). Lactation 1W (LD 0-7): total n=464 (n=446[130], n=18[131]). Lactation 2W (LD 8-14): total n=258 (n=222[130], n=36[132]). Lactation 3W (LD 15-21): total n=260 (n=224[130], n=36[131]). Lactation 4W+ (LD 21+): total n=33 (n=18[131], n=15[132]). Dry period 2W pre (0-14d parturition): total n=258 (n=205[130], n=36[131], n=36[133]). Dry period 4W pre (15-28d prepartum): total n=258 (n=18[131], n=10[134], n=3[135]). “-” indicates that the value was not reported in the original source. All values are averages of data from published literature. All dairy cows were Holstein-Friesian. PND: postnatal day; LD: lactation day; HF: Holstein Friesian cows; B. t. indicus: Bos taurus indicus.

**Table 3:** Plasma free amino acid concentrations across different physiological stages in pigs.

AA (μM)	Pre-weaned	Nursery^a^	Puberty^a^	Adult^a^	Gestation					Lactation	
					Early	Mid	Late		Early	Mid	Late
Ile	156.69	137.01	185.03	102.87	117.33	107.75	125.53		99.70	98.00	112.52
His	81.94	86.75	100.55	87.30	81.50	84.00	97.35		80.80	74.33	89.48
Met	68.92	33.04	65.28	48.92	45.33	48.13	66.87		48.18	42.44	62.09
Val	339.93	205.47	422.11	292.03	325.67	286.25	307.30		243.37	279.22	289.54
Lys	262.18	297.72	273.75	280.91	259.00	274.25	315.27		184.20	131.22	180.24
Leu	188.83	243.45	257.68	245.29	217.33	205.38	240.80		146.50	176.11	191.07
Trp	35.62	66.91	55.82	68.19	64.50	65.00	58.70		33.99	47.89	37.56
Phe	99.23	97.18	142.50	107.91	77.67	80.00	82.60		112.00	77.00	97.64
Thr	211.36	446.01	363.50	145.95	147.33	149.25	155.10		111.70	109.33	202.60
Arg	83.73	145.65	309.53	225.15	163.67	175.13	286.77		164.33	128.33	135.58
Gly	975.22	1362.67	1333.29	1014.32	769.00	915.88	898.30		767.70	755.56	1065.94
Ser	207.22	195.68	318.96	145.32	133.67	159.13	166.70		84.60	115.11	143.55
Tyr	117.76	115.76	139.52	106.68	84.33	89.50	103.07		82.20	64.78	100.80
Glu	165.51	228.89	461.63	211.98	107.00	172.50	171.95		120.30	158.33	148.24
Gln	530.40	-	738.40	283.48	516.00	464.13	421.53		530.50	481.56	444.69
Ala	592.74	656.74	1322.11	574.27	473.00	584.63	859.83		382.73	447.44	509.29
Pro	336.42	-	810.91	239.67	267.00	295.75	356.77		286.33	224.00	258.20
Asp	27.80	153.5	39.29	26.92	4.50	8.00	16.60		18.90	35.67	23.25
Asn	81.80	-	99.53	64.48	40.00	44.50	-		58.00	51.50	84.66
Cys	77.54	17.54	-	-	79.50	50.00	2.14		92.83	136.00	72.32
Tau	173.00	-	-	151.63	56.00	78.13	81.60		88.80	116.84	47.13
Cit	109.84	-	-	77.00	81.00	96.00	106.30		89.80	69.67	79.74
Orn	104.70	-	-	89.03	84.33	117.75	159.00		82.53	67.00	69.23

^a^ Data from serum; all other values are from plasma. Pre-weaned (PND 0-29, 0-6 kg): total n=110; YL (n=10[136], n=60[137], n=20^138^), DLY (n=20)[139] and our unpublished data (n=6). Nursery (PND 30-70, 6-30 kg): DLY (n=6)[140]. Puberty (PND 70-120, 30-60 kg): LHD (n=6)[141]. Adult (PND 120-180, 60-100 kg): total n=32; DLY (n=6)[142] and our unpublished data (n=28). Gestation Early (GD 0-40): total n=36; GL (n=18)[143] and YL (n=18)[144]. Gestation Mid (GD 41-80): total n=54; GL (n=18)[145] and YL (n=18^144^, n=18[145]). Gestation Late (GD 81-110): total n=34; YL (n=26[144], n=8[145]). Lactation Early (LD 0-7): total n=27; YL (n=10[145], n=10[137], n=7[146]). Lactation Mid (LD8-15): total n=26; YL (n=10[137], n=10[146]) and our unpublished data(n=6). Lactation Late (LD 16-29): total n=48; YL ((n=10[137], n=9[146], n=6^147^), GL (n=12)[148] and our unpublished data(n=11). “-” indicates that the value was not reported in the original source. All data are averages of values from literature review and our own research. Our unpublished data is from DLY pigs. PND: postnatal day; GD: gestational day; LD: lactation day; YL: Yorkshire × Landrace pigs; DLY: Duroc × Landrace × Yorkshire pigs; LHD: Landrace × Hampshire × Duroc pigs; GL: German Landrace pigs

**Table 4:** Free amino acid content in colostrum and mature milk across different mammalian species and physiological stages.

AA(μM)	Human			Mice		Cow			Pig	
	Colostrum	Mature		Mature		Colostrum	Mature		Colostrum	Mature
Ile	314.98	292.27		11.97		138.50	1.26		6.41	16.28
His	112.48	115.82		9.00		8.34	0.98		557.79	400.74
Met	73.69	139.89		3.79		5.49	1.41		5.99	17.76
Val	481.34	450.87		65.85		474.55	32.14		42.70	91.36
Lys	337.34	325.75		71.20		15.64	21.70		22.40	56.55
Leu	605.15	576.34		11.48		30.20	7.45		24.10	37.61
Trp	4.22	4.51		5.18		2.97	0.74		6.42	16.99
Phe	180.49	197.13		6.00		8.02	0.88		18.09	32.05
Thr	363.61	515.60		47.25		13.71	2.17		54.09	203.49
Arg	159.11	402.95		67.00		6.01	3.18		27.97	67.89
Gly	349.55	414.68		298.55		17.10	5.61		169.31	790.02
Ser	417.70	390.72		196.95		2.16	2.41		35.47	246.50
Tyr	137.37	226.00		4.05		10.14	7.97		15.38	49.10
Glu	942.11	1461.21		189.00		67.28	359.5		218.33	846.55
Gln	2580.03	174.22		10.10		20.76	31.21		184.60	2051.74
Ala	657.62	526.59		265.00		117.89	63.02		139.04	458.89
Pro	695.08	604.56		87.00		54.45	-		34.37	88.40
Asp	440.05	421.54		31.75		-	-		95.44	399.45
Asn	20.56	23.09		14.30		-	-		29.64	176.47
Cys	34.33	61.88		10.00		-	-		93.21	295.03
P-Ser	-	39.07		-		-	-		60.57	-
Tau	169.85	223.52		425.00		848.33	97.16		1069.79	1302.92
Cit	15.45	17.75		-		2.11	9.24		6.26	37.78
Orn	5.64	10.46		4.58		32.39	16.03		25.51	48.63

Human Colostrum (days 1-5 postpartum): total n=33 (n=3^149^ and n=30^150^). Human Mature (>5 days postpartum): total n=223 (n=30^150^ and n=193^151^). Mice Mature (>3days postpartum): total n=11 (n=5^152^ and n=6^127^. Cow Colostrum (days 1-3 postpartum): total n=30 (n=20^153^ and n=10^154^). Cow Mature (>3days postpartum): total n=30 (n=20^153^ and n=10^154^). Pig Colostrum (days 1-3 postpartum): total n=32 (n=10^143^, n=10^155^, n=6^156^ and our unpublished data (n=6). Pig Mature (>3days postpartum): total n=56 (n=20^137^, n=20^155^, n=6^156^, n=10^157^). “-” indicates that the value was not reported in the original source. All values are averages of data from published literature and our own research. All pigs were YL crossbred (Yorkshire × Landrace).

## Abbreviations

TEBs: terminal end buds
ECM: extracellular matrix
IGF-1: Insulin like growth factor
EGF: Epidermal growth factor
TGF-β: Transforming growth factor beta
AAs: Amino acids
mTOR: mechanistic target of rapamycin
ERα: estrogen receptor alpha
GH: growth hormone
RANK: receptor activator of nuclear factor kappa B
PRLR: Prolactin receptor
JAK2: Janus kinase
STAT5: signal transducer and activator of transcription
LumHR: uminal hormone responsive
AREG: amphiregulin
HB-EGF: EGF like growth factor
EGFR: epidermal growth factor receptor
FGF10: fibroblast growth factor 10
FGFR2b: fibroblast growth factor receptor 2b
HGF: hepatocyte growth factor
MMPs: matrix metalloproteinases
FAK: focal adhesion kinase
YAP: Yes associated protein
TAZ: transcriptional coactivator with PDZ binding motif
MaSCs: Mammary stem cells
DDR1: discoidin domain receptor 1
RUNX1: runt related transcription factor
MECs: Mammary epithelial cells
VANGL2: vang like protein
RTK: receptor tyrosine kinase
HA: hyaluronic acid
STAT3: signal transducer and activator of transcription 3
NF-κB: nuclear factor kappa-light-chain-enhancer of activated B cells
GLUT1: glucose transporter type 1
PUFAs: polyunsaturated fatty acids
DHA: docosahexaenoic acid
GPR120: G protein-coupled receptor 120
cAMP: cyclic adenosine monophosphate
EPAC: exchange protein directly activated by cAMP
CD36: cluster of differentiation 36
JNK: c-Jun N-terminal kinase
ERK: extracellular signal-regulated kinase
ERK1/2: extracellular signal regulated kinase
CDK1: cyclin dependent kinase
AhR: hydrocarbon receptor
BDNF: brain derived neurotrophic factor
TLR2: Toll like receptor
TLR4: Toll like receptor
ATG: autophagy related
IGF-1R: insulin like growth factor 1 receptor
CX3CL1: C-X3-C motif chemokine ligand 1, also known as fractalkine
MHCII: major histocompatibility complex class II
IL-6: interleukin 6
TNF-α: tumor necrosis factor α
PI3K: phosphatidylinositol 3 kinase
C/EBPβ: CCAAT/enhancer-binding protein beta
IFN-γ: interferon gamma
liMacs: Lactation induced macrophages
CSF-1: colony stimulating factor
IGFBP-3: insulin-like growth factor-binding protein 3
MCP-1: monocyte chemoattractant protein 1
MAdCAM-1: mucosal addressin cell adhesion molecule 1
CCL28: C C motif chemokine ligand
GM-CSF: granulocyte-macrophage colony-stimulating factor
NET: neutrophil extracellular trap
CCL2: C C motif chemokine ligand
CXCL1: C-X-C motif chemokine ligand 1
EAAC1: excitatory amino acid carrier 1
GLAST: glutamate aspartate transporter
iNOS: inducible nitric oxide synthase
EMPs: erythro myeloid progenitors
GnRH: gonadotropin releasing hormone
GS: glutamine synthetase
NO: nitric oxide
TDO: tryptophan 2,3 dioxygenase
IDO1/2: indoleamine 2,3 dioxygenase
GCN2: general control nonderepressible 2
5-HIAA: 5 hydroxyindoleacetic acid
BCAT: branched chain aminotransferase
BCKAs: branched chain ketoacids
BCKAD: branched chain ketoacid dehydrogenase
